# Can patients screen themselves? Pilot study of an audio-guided computer-assisted self-interview (ACASI) approach to screening for substance use in primary care

**DOI:** 10.1186/1940-0640-7-S1-A7

**Published:** 2012-10-09

**Authors:** Jennifer McNeely, Brian Gilberti, Rubina Khan, John Rotrosen, Shiela Strauss, Marc Gourevitch

**Affiliations:** 1 Department of Medicine, New York University School of Medicine, New York, NY, USA; 2 Wagner School of Public Service, New York University, New York, NY, USA; 3 Department of Psychiatry, New York University School of Medicine, New York, NY, USA; 4 New York University College of Nursing, New York, NY, USA; 5 Department of Population Health, New York University School of Medicine, New York, NY, USA

## 

Lack of a brief, validated screening and assessment tool to identify problematic drug use is a significant barrier to integrating screening, brief intervention and referral to treatment (SBIRT) services into primary care settings. Because patient self-administered screening is potentially more efficient than the traditional face-to-face approach, we undertook a pilot study examining the feasibility and acceptability of an audio-guided computer-assisted self-interview (ACASI) to identify substance use. We adapted the World Health Organization’s Alcohol, Smoking, and Substance Involvement Screening Test (ASSIST) to ACASI format and administered it on touch-screen tablet computers. English- and Spanish-speaking patients were recruited from a large urban primary care clinic. Participants completed the ACASI ASSIST in the waiting area and received a $4.50 transit card. Of 47 eligible patients approached, 35 (74%) agreed to participate. Participants were 57% male with a mean age of 49 years (range 28–72 years, SD = 11). The majority (54%) were foreign born, 50% were Hispanic, and 29% were African American. Twenty-five subjects completed the ASSIST in English, and 10 in Spanish. Thirty participants (86%) screened positive for lifetime use of alcohol, and 18 (51%) for other drugs (excluding tobacco). Twenty-two (63%) had used alcohol and/or other drugs in the past three months, and 13 (37%) had moderate- or high-risk use (6 alcohol; 11 other drugs; 4 both). Mean time to complete the ACASI ASSIST was 5.6 minutes (range, 1.5–17.2 minutes, SD = 3.2). Responses were 100% complete. All but one participant felt comfortable answering these questions on a computer. Most either preferred the computer to an interviewer (50%) or had no preference (38%). These results indicate that computer-assisted substance use screening may be feasible and acceptable among a culturally diverse primary care patient population. Our next step will be to evaluate the validity of the ACASI-administered ASSIST.

